# Robustness of hypofractionated breast radiotherapy after breast-conserving surgery with free breathing

**DOI:** 10.3389/fonc.2023.1259851

**Published:** 2023-10-31

**Authors:** Kunzhi Chen, Wuji Sun, Tao Han, Lei Yan, Minghui Sun, Wenming Xia, Libo Wang, Yinghua Shi, Chao Ge, Xu Yang, Yu Li, Huidong Wang

**Affiliations:** ^1^ Department of Radiation Oncology & Therapy, The First Hospital of Jilin University, Changchun, China; ^2^ Jilin Provincial Key Laboratory of Radiation Oncology & Therapy, Department of Radiation Oncology & Therapy, The First Hospital of Jilin University, Changchun, China; ^3^ NHC Key Laboratory of Radiobiology, School of Public Health, Jilin University, Changchun, China

**Keywords:** robustness, breast cancer, hypofractionated radiotherapy, skin flash tool, hybrid planning

## Abstract

**Purpose:**

This study aimed to evaluate the robustness with respect to the positional variations of five planning strategies in free-breathing breast hypofractionated radiotherapy (HFRT) for patients after breast-conserving surgery.

**Methods:**

Twenty patients who received breast HFRT with 42.72 Gy in 16 fractions were retrospectively analyzed. Five treatment planning strategies were utilized for each patient, including 1) intensity-modulated radiation therapy (IMRT) planning (IMRT_pure_); 2) IMRT planning with skin flash tool extending and filling the fluence outside the skin by 2 cm (IMRT_flash_); 3) IMRT planning with planning target volume (PTV) extended outside the skin by 2 cm in the computed tomography dataset (IMRT_ePTV_); 4) hybrid planning, i.e., 2 Gy/fraction three-dimensional conformal radiation therapy combined with 0.67 Gy/fraction IMRT (IMRT_hybrid_); and 5) hybrid planning with skin flash (IMRT_hybrid-flash_). All plans were normalized to 95% PTV receiving 100% of the prescription dose. Six additional plans were created with different isocenter shifts for each plan, which were 1 mm, 2 mm, 3 mm, 5 mm, 7 mm, and 10 mm distally in the X (left-right) and Y (anterior-posterior) directions, namely, (X,Y), to assess their robustness, and the corresponding doses were recalculated. Variation of dosimetric parameters with increasing isocenter shift was evaluated.

**Results:**

All plans were clinically acceptable. In terms of robustness to isocenter shifts, the five planning strategies followed the pattern IMRT_ePTV_, IMRT_hybrid-flash_, IMRT_flash_, IMRT_hybrid_, and IMRT_pure_ in descending order. *V*_95%_ of IMRT_ePTV_ maintained at 99.6% ± 0.3% with a (5,5) shift, which further reduced to 98.2% ± 2.0% with a (10,10) shift. IMRT_hybrid-flash_ yielded the robustness second to IMRT_ePTV_ with less risk from dose hotspots, and the corresponding *V*_95%_ maintained >95% up until (5,5).

**Conclusion:**

Considering the dosimetric distribution and robustness in breast radiotherapy, IMRT_ePTV_ performed best at maintaining high target coverage with increasing isocenter shift, while IMRT_hybrid-flash_ would be adequate with positional uncertainty<5 mm.

## 1 Introduction

Over the past decades, adjuvant post-operative radiotherapy has been the standard treatment for patients with breast cancer receiving breast-conserving surgery to reduce local recurrence (1–3). Several clinical trials have shown that moderately hypofractionated radiotherapy (HFRT), typically delivered with a dose of 40–42.72 Gy in 15–16 fractions, provides equivalent tumor control, long-term toxicity, and cosmetic outcome compared with prolonged conventionally fractionated radiotherapy with a standard dose of 50–50.4 Gy in 25–28 fractions (4–9). Hypofractionation schedules reduce the fraction number and overall treatment period, which is beneficial for patients and treatment providers (9, 10).

Intensity-modulated radiation therapy (IMRT) is utilized in treating breast cancer for its high target dose coverage and homogeneity. However, the dosimetric effects of inter- and intra-fraction positioning variations have always been a concern in highly modulated fields owing to the challenging patient setup and the anatomical variations caused by respiratory motion, breast shape change, and edema (11–13). Various imaging guidance and motion management techniques, such as deep inspiration breath hold (14–16), continuous positive airway pressure (14), four-dimensional computed tomography (11), cone-beam computed tomography (17), and surface tracking (15, 16, 18), have been developed to limit dosimetric error. Nonetheless, these techniques could be physically challenging for some patients, and the dosimetric error can only be alleviated but not eliminated. Therefore, the robustness of treatment delivery should be taken into account during treatment planning, especially for HFRT with free breathing, considering that the potential hazard from one fraction could bring a rather substantial effect to the treatment with fewer fractions.

Several techniques have been developed over the years to assure the robustness of breast radiotherapy, including skin flash (19–21), planning target volume (PTV) extension, which is also known as the “virtual bolus” technique (21–23), and hybrid planning (24, 25). The skin flash technique uses a flash region consisting of an extended irradiation volume outside the skin to account for positional variations, which can be achieved in several ways, such as using the skin flash tool integrated into the Eclipse treatment planning system (TPS; Varian Medical Systems, Palo Alto, CA, USA). The PTV extension technique involves the modification of the computed tomography (CT) dataset by extending the PTV outside the skin (21). Treatment plans would be optimized based on the modified CT data and subsequently copied and recalculated onto the original CT dataset (19–21). The hybrid planning technique consists of a combination of IMRT and three-dimensional conformal radiation therapy (3D-CRT) (24, 25). Positional and anatomical variations are accounted for by manually opening the jaw and using a multi-leaf collimator (MLC) for 3D-CRT fields.

Previous studies have discussed the importance of robustness and the efficiency of some techniques in assuring adequate target coverage during irradiation (21). However, to our knowledge, the dosimetric and robustness-wise differences among the above planning strategies remain unclear, especially for HFRT. This study aimed to compare the dosimetric characteristics and robustness with respect to positional variations among five planning strategies for free-breathing breast HFRT.

## 2 Materials and methods

**Figure 1:**
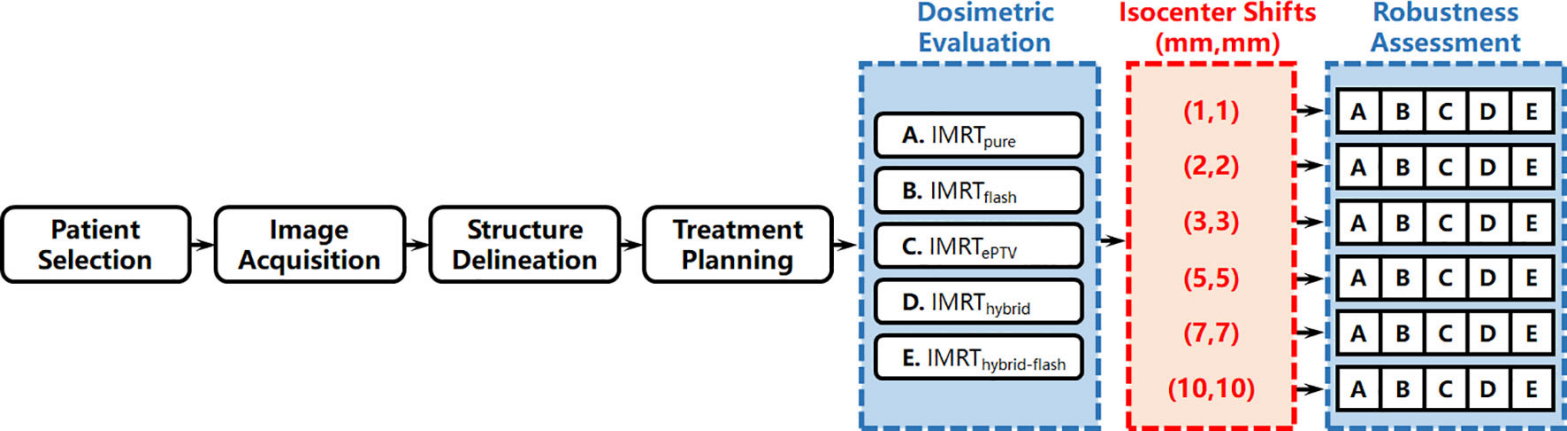
Overall workflow of this study.

The workflow of this study is summarized in **Figure 1** and detailed as follows.

### 2.1 Patient selection and simulation

Twenty patients with breast cancer (10 on the left side and 10 on the right side) treated with HFRT after breast-conserving surgery were retrospectively analyzed in this study. Prophylactic supraclavicular irradiation and partial simultaneous integrated boost were not involved for all patients. Patients were between 35 and 76 years of age with a median of 50 years.

Patients were immobilized in a supine position with a CIVCO breast board (CIVCO Medical Solutions, Coralville, IA, USA). CT simulation was performed for all patients with free breathing on a Philips Brilliance Big Bore CT scanner (Philips Healthcare, Cleveland, OH, USA) with a slice thickness of 5 mm. The CT datasets were subsequently transferred to the Varian Eclipse TPS version 15.6 (Varian Medical Systems, Palo Alto, CA, USA).

### 2.2 Target definition

According to the National Comprehensive Cancer Network guidelines (2018) (26), Report No. 9804 of the Radiation Therapy Oncology Group (27, 28), and the ESTRO consensus guideline (29, 30), the clinical target volume (CTV) was contoured in the TPS, including intact breast tissue and tumor bed on the ipsilateral side. The PTV was defined by adding an isotropic 5-mm margin to the CTV. The PTV was limited posteriorly to the lung surface and anteriorly within 4 mm from the skin. Subsequently, the organs at risk (OARs) were contoured, including the lung, heart, and liver. The breast target volume of each patient is summarized in **Supplementary Table 1.**

### 2.3 Treatment planning

A total dose of 42.72 Gy in 16 fractions (i.e., 2.67 Gy per fraction) was prescribed for the PTV of all patients. Treatment plans were optimized with Photon Optimizer (version 15.6) in Eclipse TPS for a Varian TrueBeam linear accelerator equipped with a 120-leaf Millennium MLC using 6-MV beams. The maximum dose rate was 600 MUs/min. The AXB algorithm version 15.6 was used for dose calculation with a 2.5-mm grid.

For each patient, five treatment plans were created using different strategies, and the initial optimization parameters were kept the same, as well as the following plan fine-tuning. All plans were normalized to 95% of the PTV receiving 100% of the prescription dose. All plans met the dose–volume constraints based on the guidelines from the National Cancer Center and the National Cancer Quality Control Center of China (31). All plans can be considered clinically acceptable. The main constraints were summarized as follows: PTV *V*_110%_< 5% and *V*_107%_< 15%; ipsilateral lung *D*_mean_< 10 Gy, *V*_20Gy_< 20%, and *V*_5Gy_< 40%; heart *D*_mean_< 10 Gy (left-side breast cancer). The adopted planning strategies are summarized as follows.

**Figure 2:**
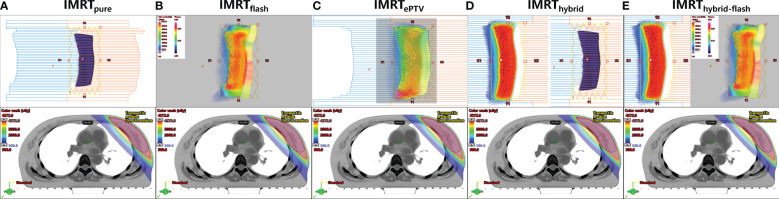
Technical features of five planning strategies (upper panel) and corresponding dosimetric distributions for one patient (lower panel). **(A)** IMRT_pure_, **(B)** IMRT_flash_, **(C)** IMRT_ePTV_, **(D)** IMRT_hybrid_, **(E)** IMRT_hybrid-flash_.

1) IMRT planning (IMRT_pure_). The IMRT plan consisted of four or six fields depending on the thickness of the breast tissue. The technical feature is shown in the upper panel of **Figure 2A**.
2) IMRT planning with skin flash (IMRT_flash_). The technical feature is shown in the upper panel of **Figure 2B**. After plan optimization, the fluence of each field was extended outside the skin by 2 cm with the skin flash tool in the Eclipse TPS. Regarding the skin flash tool settings, the “nearest cell” fill method was used with the brush size set at 20 mm, the brush ceiling set at 0.01, and the cut range set at 7 mm.
3) IMRT planning with extended PTV (referred to as IMRT_ePTV_). The “PTV+2” structure was defined by extending the PTV by 2 cm outside of the body. The region of PTV+2 excluding the body was assigned a Hounsfield unit (HU) equal to the average HU of the PTV. Based on this modified CT dataset, an IMRT plan was created and optimized on the PTV+2 with the same parameters as IMRT_pure_. This plan was subsequently copied to the original CT dataset and recalculated. The technical feature is shown in the upper panel of **Figure 2C**.
4) Hybrid planning (IMRT_hybrid_). It is the combination of a 2 Gy/fraction 3D-CRT plan and a 0.67/fraction IMRT plan. The 3D-CRT plan consisted of two tangential fields with the MLC manually opened by 2 cm outside the body. The IMRT plan consisted of two or four fields depending on the thickness of the breast tissue. The technical feature is shown in the upper panel of **Figure 2D**.
5) Hybrid planning with skin flash (IMRT_hybrid-flash_). This plan was obtained by extending the fluence of each IMRT field in IMRT_hybrid_ outside the skin by 2 cm using the skin flash tool. The technical feature is shown in the upper panel of **Figure 2E**.

### 2.4 Plan comparison

The dose distributions of the five planning strategies were compared using the following parameters: conformity index (CI), mean dose (*D*_mean_) to PTV, maximum dose to 2 cc of PTV (*D*_2cc_), the percentage of PTV receiving 107% of the prescription dose (*V*_107%_), mean dose to the heart (left-side breast cancer) or liver (right-side breast cancer), mean dose to the ipsilateral lung, and the percentage of lung receiving 5 Gy (*V*_5_), 10 Gy (*V*_10_), and 20 Gy (*V*_20_). The CI was calculated using Paddick’s formula (32, 33):


(1) $$\text{CI = }\frac{{\text{TV}}_{\text{PIV}}^{2}}{\text{TV}\times \text{PIV}},$$

where TV is the target volume, PIV is the prescription isodose volume, and TV_PIV_ is the target volume within the prescription isodose volume.

### 2.5 Robustness assessment

**Figure 3:**
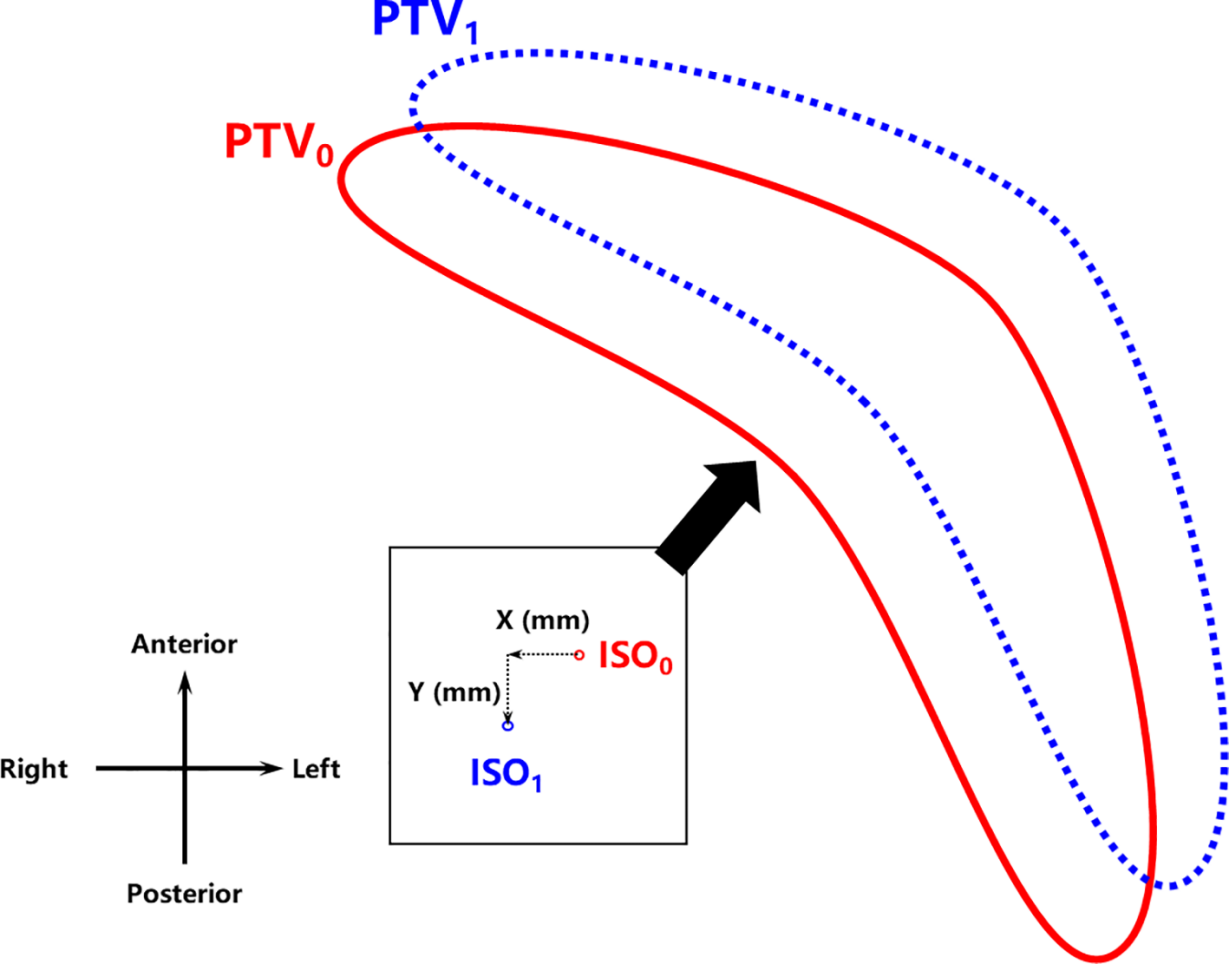
Isocenter shift geometry for robustness assessment. ISO_0_ and ISO_1_ refer to the isocenter whereas PTV_0_ and PTV_1_ refer to the relative positions of the PTV before and after the isocenter shift (X,Y), respectively. PTV, planning target volume.

Isocenter shifts were performed to simulate the error in radiotherapy delivery due to the free breathing of the patient in the TPS to assess plan robustness as demonstrated in **Figure 3**. Simultaneous shifts of 1 mm, 2 mm, 3 mm, 5 mm, 7 mm, and 10 mm distally in the X (left–right (LR)) and Y (anterior–posterior (AP)) directions, namely, (1,1), (2,2), (3,3), (5,5), (7,7), and (10,10), were applied for each set of plans. That is, for each planning strategy of each patient, six additional plans with different isocenter shifts were created and recalculated to compare with the original plan. Dosimetric parameters, including PTV *V*_100%_, *V*_95%_, CI, and mean doses to the PTV, lung, liver, and heart, were compared among the six sets of plans with increasing isocenter shifts for the plan robustness assessment.

### 2.6 Data analysis and statistics

All data are shown as mean ± standard deviation (SD). The Friedman test was used for the comparison of dosimetric parameters among five sets of plans. *p*< 0.05 was defined as statistically significant. If statistical significance was found in the Friedman test, the Bonferroni–Dunn test was used for *post-hoc* analysis. The corresponding *p*-values were evaluated with Bonferroni correction; i.e., *p*< 0.005 was defined as statistically significant. IBM SPSS Statistics 23.0 software (IBM Corporation, Armonk, NY, USA) was used for statistical analyses.

## 3 Results

The dosimetric distributions of the five planning strategies for one typical patient are shown in the lower panel of **Figure 2**. The dosimetric parameters of all plans and the corresponding Friedman test results are shown in **Table 1**. The *post-hoc* pairwise analysis results for the Friedman tests are included in **Supplementary Figure 2**. All plans generated were clinically acceptable. Significant differences were found for most examined parameters among the five sets of plans (*p*< 0.05). The average PTV mean dose, *D*_2cc_, and *V*_107%_ of the IMRT_ePTV_ plans were the highest among all plans. However, the liver mean dose and lung-associated parameters were the lowest in IMRT_ePTV_ plans.

**Table 1:** Dosimetric comparison of five planning strategies without isocenter shift, including the corresponding *p* of Friedman tests. .

Structure	Parameter	IMRT_pure_	IMRT_flash_	IMRT_ePTV_	IMRT_hybrid_	IMRT_hybrid-flash_	Friedman *p*
**PTV**	*D*_mean_ (cGy)	4,379.76 ± 23.75	4,409.34 ± 39.13	4,452.72 ± 24.14	4,401.14 ± 14.21	4,392.72 ± 40.84	<0.001
	CI	0.78 ± 0.03	0.78 ± 0.03	0.76 ± 0.03	0.75 ± 0.03	0.74 ± 0.03	<0.001
	*D*_2cc_ (cGy)	4,515.97 ± 50.05	4,539.65 ± 65.58	4,628.51 ± 53.40	4,534.4 ± 41.59	4,557.08 ± 34.53	<0.001
	*V*_107%_ (%)	0.13 ± 0.3	0.21 ± 0.35	3.02 ± 1.67	0.06 ± 0.19	0.09 ± 0.17	<0.001
**Heart** ^*^	*D*_mean_ (cGy)	265.22 ± 186.14	269.52 ± 160.02	268 ± 159.97	267.69 ± 175.38	271.94 ± 175.37	0.745
**Liver** ^†^	*D*_mean_ (cGy)	287.57 ± 143.16	298.83 ± 146.23	263.41 ± 133.45	292.48 ± 142.02	293.26 ± 141.22	<0.001
**Lung**	*V*_5_ (%)	33.54 ± 5.86	34.65 ± 6.24	32.44 ± 4.99	33.58 ± 5.31	34.01 ± 5.38	<0.001
	*V*_10_ (%)	23.89 ± 5.25	24.63 ± 5.42	22.29 ± 3.72	24.14 ± 4.59	24.4 ± 4.62	0.015
	*V*_20_ (%)	15.36 ± 4.4	15.56 ± 4.45	13.97 ± 2.98	15.78 ± 3.62	15.9 ± 3.66	<0.001
	*D*_mean_ (cGy)	735.12 ± 111.8	743.04 ± 112.58	708.02 ± 98.21	781.2 ± 126.6	792.89 ± 132.06	<0.001

The parameters are shown as mean ± SD.

IMRT, intensity-modulated radiation therapy; PTV, planning target volume; CI, conformity index.

^*^ For 10 patients with left-side breast cancer.

^†^ For 10 patients with right-side breast cancer.

**Figure 4:**
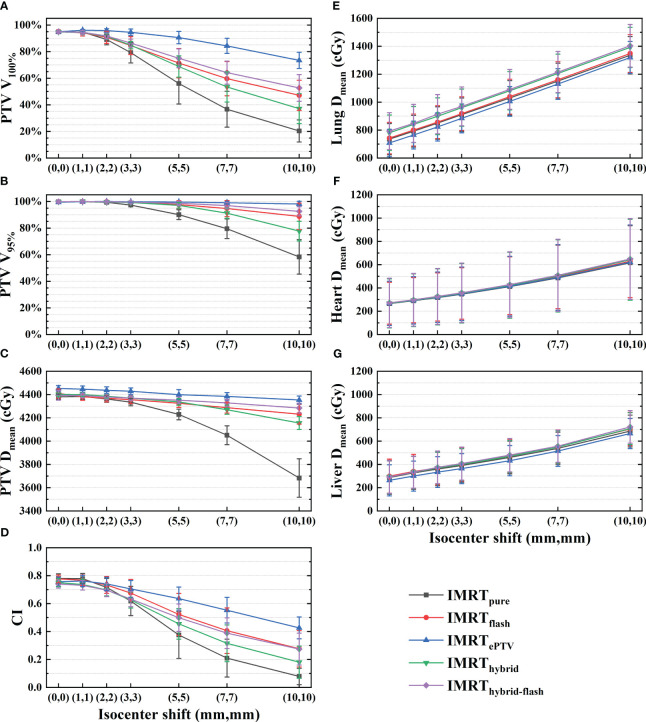
Variations in **(A)** *V*_100%_, **(B)** *V*_95%_, **(C)** PTV mean dose, **(D)** CI, **(E)** lung mean dose, **(F)** heart mean dose, and **(G)** liver mean dose with increasing isocenter shift for the five planning strategies. PTV, planning target volume; CI, conformity index.

A demonstration of the dose distribution variation among five plans with increasing isocenter shift can be found in **Supplementary Figure 1**. Variations of the PTV mean dose, target coverage, and OAR mean doses (mean ± standard deviation) with respect to the isocenter shift are shown in **Figure 4**. In terms of target coverage and PTV mean dose, IMRT_ePTV_ exhibited the highest robustness to the isocenter shift, and then IMRT_hybrid-flash_, IMRT_flash_, IMRT_hybrid_, and IMRT_pure_ in descending order. *V*_100%_ and *V*_95%_ of IMRT_ePTV_ remained at 90.5% ± 4.6% and 99.6% ± 0.3% even with a (5,5) shift, which further reduced to 73.4% ± 6.1% and 98.2% ± 2.0% with a (10,10) shift. The mean *V*_100%_ values of the other strategies with a (1,1) shift were all over 90% but reduced to below 90% with a (3,3) shift. The mean *V*_95%_ values were above 95% for all strategies except IMRT_pure_ until (5,5), while only IMRT_ePTV_ and IMRT_hybrid-flash_ maintained this level for (7,7). For (10,10), the mean *V*_95%_ value of IMRT_ePTV_ was still maintained at a high level, which is above 95%. In addition, it should be noted that the PTV mean dose of IMRT_ePTV_ was significantly higher than that of the other plans (*p*< 0.005). Regarding CI, IMRT_flash_ outperformed IMRT_hybrid-flash_ initially but ended up at a similar level. Meanwhile, variations of OAR doses did not show any notable differences among the five sets of plans.

## 4 Discussion

The purpose of this study was to assess the robustness of five practical planning strategies for breast HFRT after breast-conserving surgery to provide information regarding the choice of treatment strategies. The dosimetric results were compared to confirm the applicability and feasibility of five strategies, and multiple isocenter shifts were used to assess the robustness of positional variations.

Previous studies have demonstrated the importance of plan robustness considerations during proton treatment planning (21). Robust optimization has shown its effectiveness in ensuring target coverage with the existence of inter- and intra-fraction motions in proton therapy (34–36). However, the usual practice for motion compensation is adding a margin to the target volume in photon therapy, and robustness is rarely considered. Nonetheless, plan robustness could potentially account for this issue in free-breathing breast radiotherapy with high positional uncertainties (21). In particular, the fraction number in HFRT is reduced from 25–28 to 15–16 compared with conventional fractionation, and the potential hazard from one fraction could bring a more substantial effect to the treatment.

Various imaging guidance and motion management techniques have been developed and utilized for free-breathing breast radiotherapy to reduce motion-induced dosimetric error. Inter- and intra-fraction positioning variations, including setup error, anatomical change, and respiratory motion, are the primary concerns for the robustness evaluation. However, inter- and intra-fraction variations cannot be eliminated with the inevitable anatomical changes. Previous studies have reported various intra- and inter-fraction variations in breast radiotherapy (14–18, 36). Lee et al. (14) reported that the median intra-fractional variations for free-breathing right-breast radiotherapy are −0.1 (range −4.2 to 3.6) and 0.6 (range −3.4 to 5.0) mm in the LR and AP directions, and the median inter-fractional variations are −0.7 (range −7.8 to 6.4) and 2.9 (range −16.9 to 9.4) mm in the LR and AP directions, respectively. In this study, considering the intra- and inter-fraction variations and the use of imaging guidance, the isocenter shift was altered from (1,1) to (10,10) for a reasonable evaluation of clinical scenarios. The isocenter shifts were not simulated in the cranial–caudal (CC) direction because the involved techniques focus on the motion compensation in the LR and AP directions. The motion in the CC direction can be accounted for by the CTV–PTV margin.

This study aimed to evaluate the robustness with respect to positional variations of five planning strategies for free-breathing breast HFRT. All plans were clinically acceptable. Based on the results in **Figure 4**, the target coverage, CI, and mean dose of the PTV for all plans decreased with increasing isocenter shift, while the OAR doses showed an increasing pattern. It can be expected since the displacement of the PTV would potentially lead to the increased exposure of OARs. In terms of PTV dosimetry with increasing isocenter shift, the five planning strategies followed the pattern IMRT_ePTV_, IMRT_hybrid-flash_, IMRT_flash_, IMRT_hybrid_, and IMRT_pure_ in descending order.

The IMRT_pure_ plans, for which no motion compensation technique was used, resulted in the worst *V*_100%_, *V*_95%_, PTV mean dose, and CI with increasing isocenter shift as expected. The IMRT_hybrid_ plans consisted of 3D-CRT and IMRT fields. The 3D-CRT fields, which contributed 75% of the prescription dose, were manually opened by 2 cm out of the body to provide plan robustness. The IMRT fields can improve target conformity and coverage, as well as OAR sparing, yet the robustness of the IMRT fields was not considered. The IMRT_flash_ technique filled the space of 2 cm outside the body with the fluence of 7 mm within the body using the skin flash tool. However, the approximated fluence was uniformly filled, and the accuracy of the PTV dose distribution with the increasing isocenter shift would be limited. IMRT_hybrid-flash_, as the combination of skin flash and hybrid planning techniques, showed better results than IMRT_hybrid_ and IMRT_flash_. The IMRT_ePTV_ plans yielded the best robustness with the use of PTV modification in the CT images and structures for plan optimization. The PTV extended outside of the body contour was assigned with a breast-tissue-equivalent HU value. The PTV coverage can be maintained at a high level because the anatomical changes can be adequately considered.

It should be noted that IMRT_ePTV_ did not behave much differently on the variation of OAR doses from the other plans, despite its higher target coverage. With the increase of isocenter shift, even though seemingly more lung, heart, and liver tissues were exposed to the radiation field, this part of the body was not substantially influenced by the applied techniques since the affected dose fluence was primarily out of the body. Therefore, there is no notable difference in the OAR doses among the five sets of plans.

Considering PTV and OAR dosimetry as a whole, the IMRT_ePTV_ yielded the best overall robustness to positional variations. However, there are downsides to the IMRT_ePTV_ strategy. The planning process is rather challenging. After the plans were optimized on the modified CT images and structures, the new plans were transferred back to the original CT dataset, and the dose was re-calculated. There were many dose hotspots (*V*_107%_ and *V*_110%_) close to the skin. In order to eliminate the dose hotspots, the dose fluence was edited manually with the fluence editing tool until the plans were accepted clinically. The IMRT_ePTV_ planning process was more complicated and time-consuming. The main reason for the emergence of dose hotspots in IMRT_ePTV_ plans is that the PTV in the final dose calculation is smaller than the PTV in the plan optimization. Although manual fluence modification could allow the plans to meet the clinical protocol, *V*_107%_ and *D*_2cc_ of the PTV were still significantly higher compared with the other strategies.

In general, the choice of planning strategies should be determined based on individual circumstances and protocols, and the extent of positional variation would vary among treatment centers depending on patients, motion management methods, and other influencers. In situations of high positional uncertainty (≥5 mm), IMRT_ePTV_ should be used to ensure high PTV coverage, but a potentially higher dose of the PTV should be carefully dealt with considering the radiation-induced breast fibrosis (37). For low positional uncertainties (<5 mm), the PTV *V*_95%_ of IMRT_hybrid-flash_ plans maintained above 95% with an isocenter shift of (5,5) as shown in **Figure 4**. Therefore, the IMRT_hybrid-flash_ strategy is recommendable since it is spared from the dosimetric disadvantage of IMRT_ePTV_ and the planning process of IMRT_hybrid-flash_ is less demanding and time-consuming.

This study has several limitations. First, the dose ratio of IMRT and 3D-CRT for the hybrid planning technique, which might influence the robustness, may not be optimal. To our knowledge, it has not been extensively investigated in previous studies. Second, the intra-fraction positioning error, which can be caused by respiratory motion, anatomical deformation, and radiation-induced tissue responses, could only reflect a local variation rather than a global variation. Therefore, the isocenter shift in this study, which may be considered a worst-case scenario, might amplify the effect of intra-fraction positioning variation in clinical practice.

## 5 Conclusions

Considering the dosimetric distributions, robustness, and amplitudes of inter- and intra-fraction positioning variations, IMRT_ePTV_ should be used to assure target coverage in situations of high positional uncertainty risk (≥5 mm), and IMRT_hybrid-flash_ would be adequate for situations with positional uncertainty<5 mm.

## Data availability statement

The original contributions presented in the study are included in the article/**Supplementary Material**. Further inquiries can be directed to the corresponding author.

## Ethics statement

Ethical approval was not required for the study involving humans in accordance with the local legislation and institutional requirements. Written informed consent to participate in this study was not required from the participants or the participants’ legal guardians/next of kin in accordance with the national legislation and the institutional requirements.

## Author contributions

KC: Conceptualization, Data curation, Methodology, Writing – original draft. WS: Data curation, Formal Analysis, Methodology, Writing – original draft. TH: Data curation, Writing – review & editing. LY: Data curation, Writing – review & editing. MS: Data curation, Writing – review & editing. WX: Data curation, Writing – review & editing. LW: Data curation, Writing – review & editing. YS: Formal Analysis, Writing – original draft. CG: Formal Analysis, Writing – review & editing. XY: Formal Analysis, Writing – review & editing. YL: Data curation, Writing – review & editing. HW: Funding acquisition, Supervision, Validation, Writing – review & editing.
