# Supplementary material for: Robustness of hypofractionated breast radiotherapy after breast-conserving surgery with free breathing
Source: Front Oncol. 2023 Oct 31;13:1259851. doi: 10.3389/fonc.2023.1259851 (PMC10644368; doi:10.3389/fonc.2023.1259851)
Supplement: Supplementary file 1 [file DataSheet_1.docx]

**Supplementary Materials**

**Supplementary Table S1.** Breast target volumes of all patients.

| **Left-side patient** | | **Right-side patient** | |
| --- | --- | --- | --- |
| **No.** | **Breast volume (cm^3^)** | **No.** | **Breast volume (cm^3^)** |
| 1 | 585.16 | 1 | 737.59 |
| 2 | 1015.93 | 2 | 1001.16 |
| 3 | 948.16 | 3 | 1590.30 |
| 4 | 513.75 | 4 | 622.12 |
| 5 | 453.87 | 5 | 771.11 |
| 6 | 361.39 | 6 | 659.68 |
| 7 | 756.92 | 7 | 755.78 |
| 8 | 1068.80 | 8 | 854.43 |
| 9 | 453.34 | 9 | 454.84 |
| 10 | 577.10 | 10 | 423.66 |


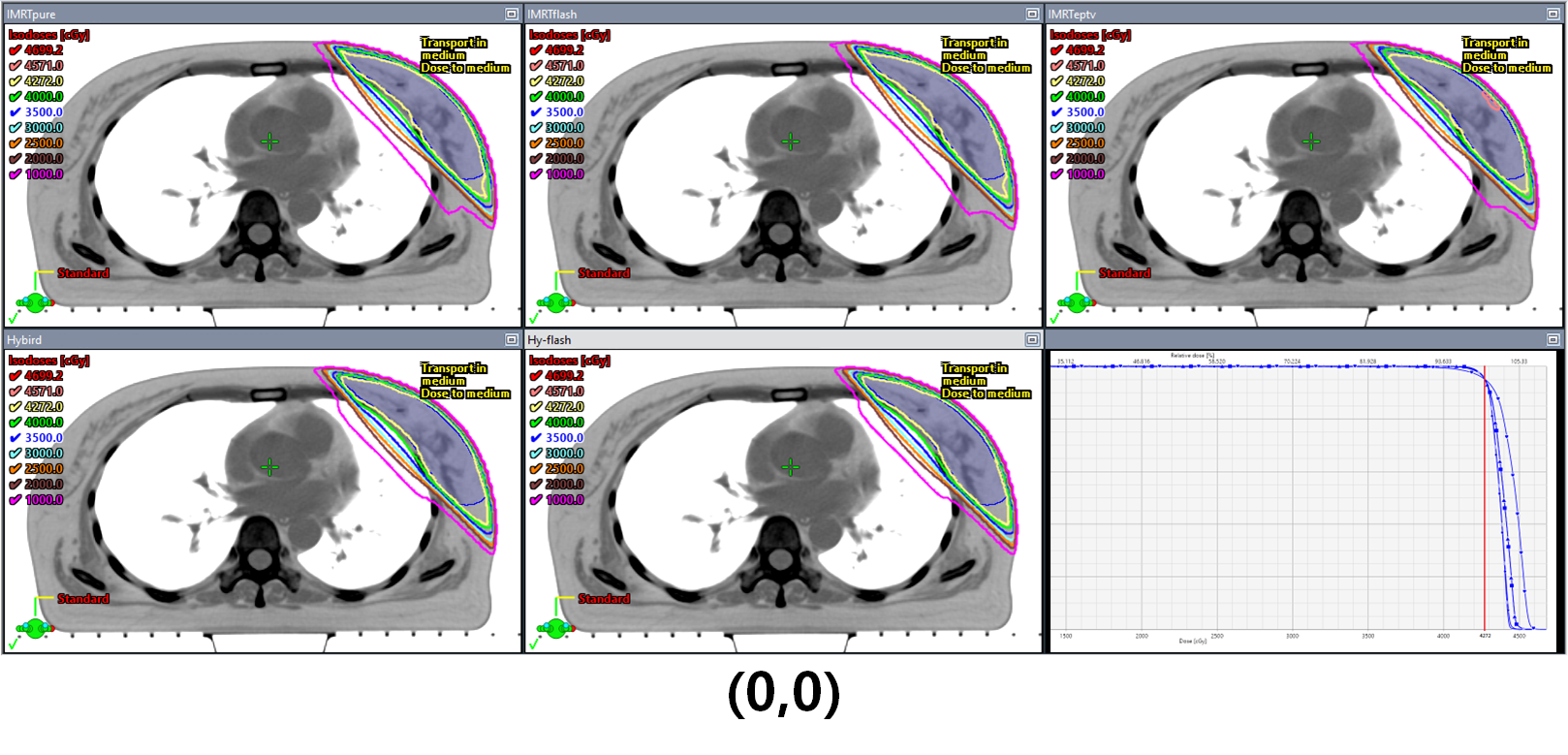


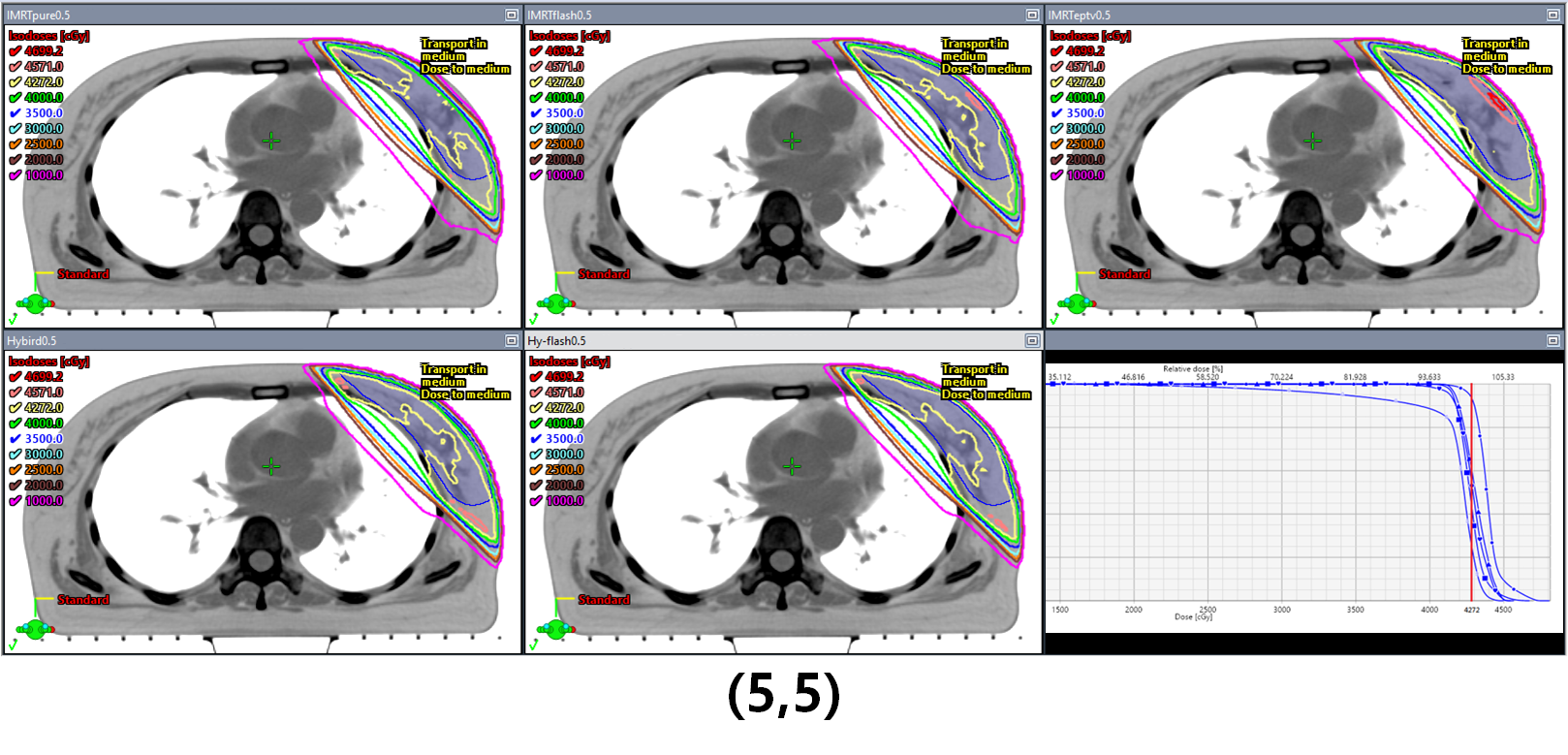


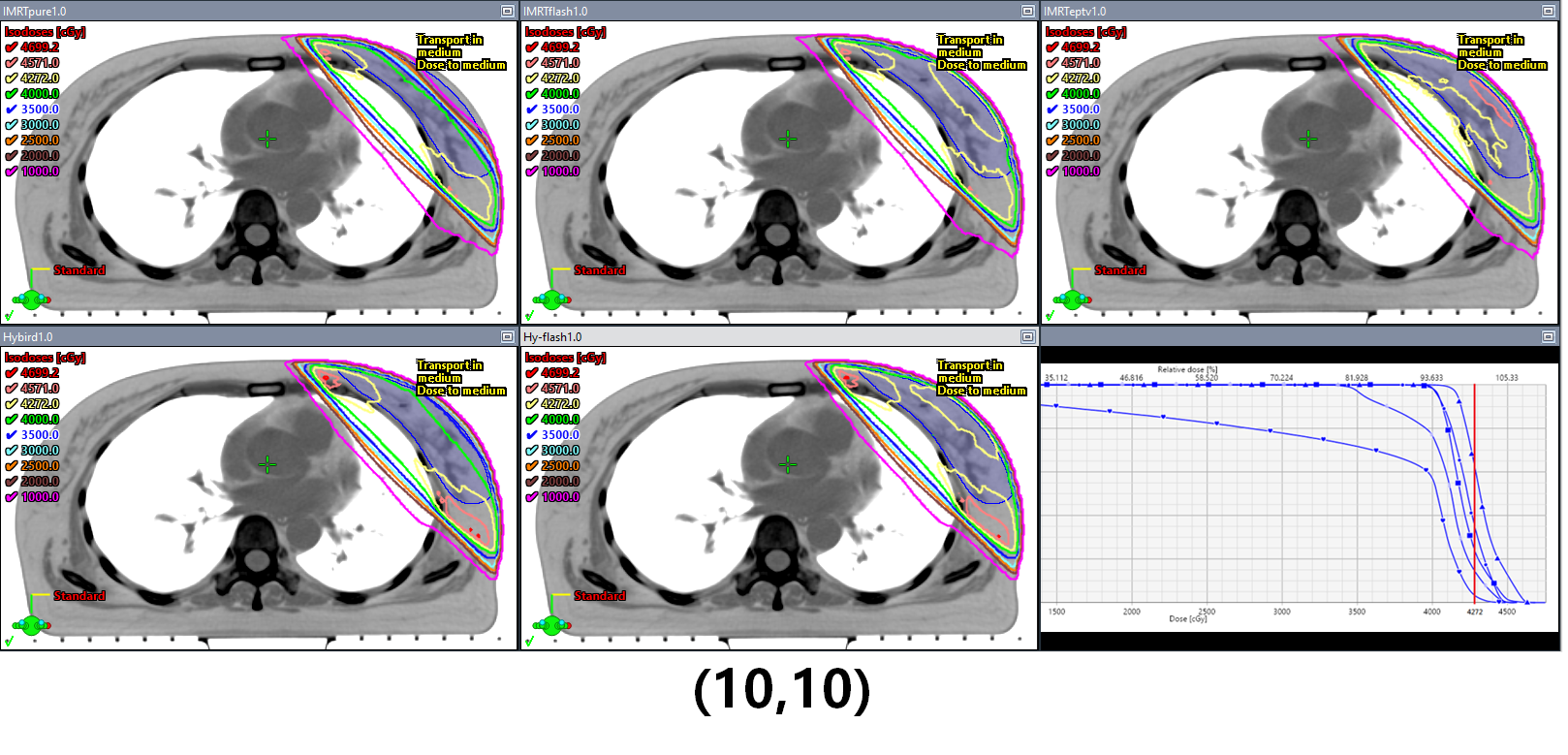


**Supplementary Figure S1.** Dose distribution comparison of IMRT_pure_, IMRT_flash_, IMRT_ePTV_, IMRT_hybrid_ and IMRT_hybrid-flash_ with (0,0), (5,5) and (10,10) isocenter shifts for one of the patients.

**
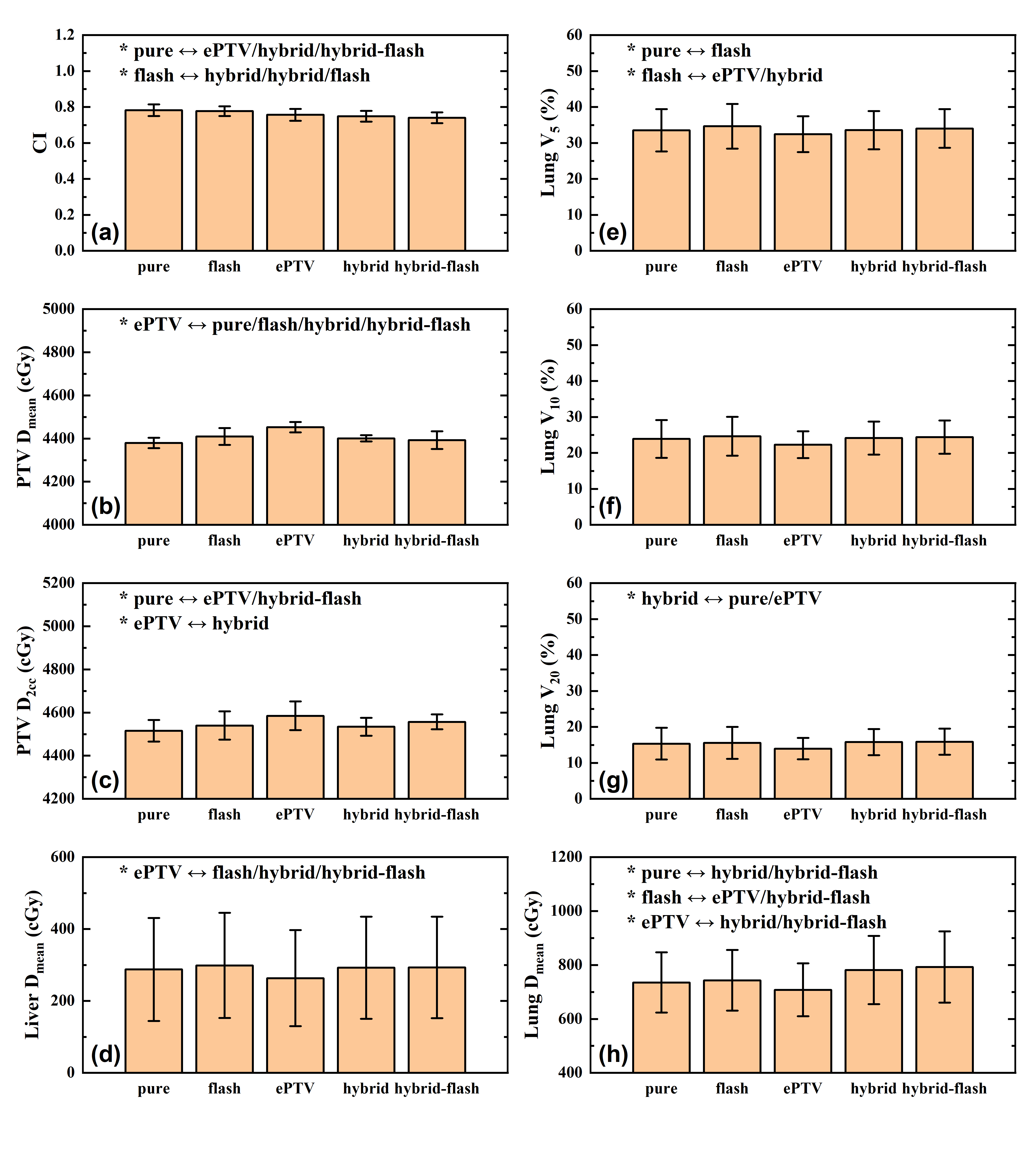
**

**Supplementary Figure S2.** Dose-volume parameters of IMRT_pure_, IMRT_flash_, IMRT_ePTV_, IMRT_hybrid_ and IMRT_hybrid-flash_ and the corresponding Bonferroni-Dunn post-hoc test results following Friedman tests. The asterisk * indicates statistically significance between listed pairs.
